# Account of Diseases in an Eastern District of London, from November 20, to December 20, 1806

**Published:** 1807-01

**Authors:** 


*Account of Diseases in an Eastern District of London, from November 20, to December 20, 1806.*

| | | | |
|---|---|---|---|
| ACUTE DISEASES. | | Fluor Albus - - - | 6 |
| Typhus - - - - - - | 7 | Epilepsia - - - | 1 |
| Scarlatina - - - - | 3 | Hysteria - - - - | 3 |
| Cynanche Tonsillaris | 2 | Paralysis - - - | 2 |
| Pneumonia - - - - | 2 | Gastrodinia - - - | 4 |
| Rheumatismus Acutus - | 3 | Diarrhœa - - - | 3 |
| CHRONIC DISEASES. | | Dysenteria - - - | 2 |
| Tussis - - - - - - | 8 | Rheumatismus Chronicus | 17 |
| Dyspnœa - - - - - | 4 | PUERPERAL DISEASES. | |
| Tussis cum Dyspnœa - | 5 | Convulsio - - - | 1 |
| Asthma - - - - - - | 1 | Ephemera - - - | 5 |
| Phthisis Pulmonalis - | 3 | Menorrhagia Lochialis | 4 |
| Hæmoptoe - - - | 2 | Mastrodinia - - - | 7 |
| Dyspepsia - - - | 5 | INFANTILE DISEASES. | |
| Anasarca - - - | 2 | Aphthæ - - - - | 4 |
| Ascites - - - - - | 1 | Ophthalmia - - - | 3 |
| Amenorrhœa - - - | 3 | Ophthalmia Purulenta | 2 |
| Menorrhagia - - | 4 | Pertussis - - - | 5 |

To the observations which were made in the last Report, respecting the prevalence of typhus, after an almost total disappearance of it, we may add that this disease still continues, and that several of its symptoms are more severe. In some cases indeed, a train of milder symptoms has appeared, and the disease has assumed more of the character of the Febris lenta of Huxham. It has approached in an insidious manner, and, instead of that rigour or violent shivering which frequently ushers in an acute disease, there has been a transient chilliness, accompanied with great lassitude, and succeeded by the other febrile symptoms. In other instances, however, after assuming the form of the simple continued fever, the disease has suddenly taken on a more alarming appearance; the sensorium has been affected, and violent delirium has supervened; the tongue and fauces have been covered with a dark coloured mucus, and petechia have appeared on different parts of the surface. Dark coloured and fœtid stools have been discharged; and to the repeated evacuations tions of this kind has been attributed the great prostration of strength which invariably attends the disease.

Attempts therefore are frequently made to stop this discharge; which, if they succeed, instead of alleviating, only tend to aggravate the malady: whereas, by permitting, or even promoting, a gradual discharge of the offensive matter, and at the same time supporting the strength of the patient, the disease is frequently brought to a favourable termination.

